# From Genotype to Phenotype—A Review of Kabuki Syndrome †

**DOI:** 10.3390/genes13101761

**Published:** 2022-09-29

**Authors:** Kelly K. Barry, Michaelangelo Tsaparlis, Deborah Hoffman, Deborah Hartman, Margaret P. Adam, Christina Hung, Olaf A. Bodamer

**Affiliations:** 1 Tufts University School of Medicine, Boston, MA 02111, USA; 2 Division of Genetics and Genomics, Boston Children’s Hospital, Boston, MA 02115, USA; 3 Takeda Pharmaceuticals, Cambridge, MA 02139, USA; 4 Orexia Therapeutics, Boston, MA 02115, USA; 5 Division of Genetic Medicine, Department of Pediatrics, University of Washington, Seattle, WA 98195, USA; 6 Broad Institute of MIT and Harvard University, Cambridge, MA 02142, USA

**Keywords:** Kabuki syndrome, Niikawa–Kuroki syndrome, transcription, KMT2D, KDM6A

## Abstract

Kabuki syndrome (KS) is a rare neuro-developmental disorder caused by variants in genes of histone modification, including *KMT2D* and *KDM6A*. This review assesses our current understanding of KS, which was originally named Niikawa–Kuroki syndrome, and aims to guide surveillance and medical care of affected individuals as well as identify gaps in knowledge and unmet patient needs. Ovid MEDLINE and EMBASE databases were searched from 1981 to 2021 to identify reports related to genotype and systems-based phenotype characterization of KS. A total of 2418 articles were retrieved, and 152 were included in this review, representing a total of 1369 individuals with KS. Genotype, phenotype, and the developmental and behavioral profile of KS are reviewed. There is a continuous clinical phenotype spectrum associated with KS with notable variability between affected individuals and an emerging genotype–phenotype correlation. The observed clinical variability may be attributable to differences in genotypes and/or unknown genetic and epigenetic factors. Clinical management is symptom oriented, fragmented, and lacks established clinical care standards. Additional research should focus on enhancing understanding of the burden of illness, the impact on quality of life, the adult phenotype, life expectancy and development of standard-of-care guidelines.

## 1. Introduction

**Figure 1:**
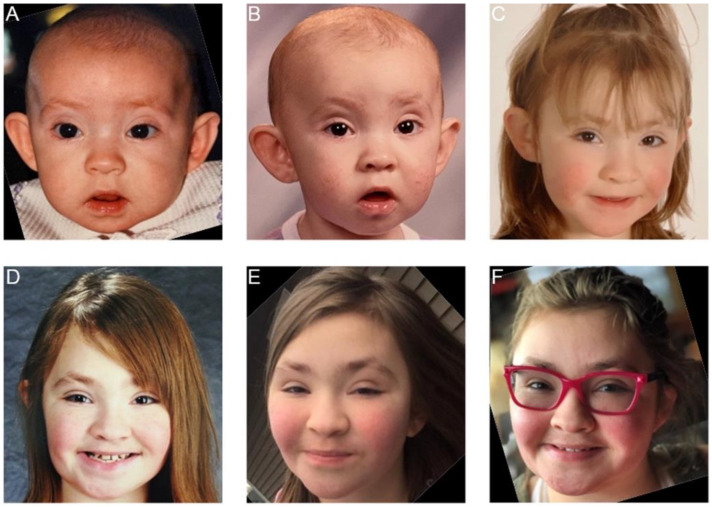
Evolution of facial features of a patient with a pathogenic variant in *KMT2D* at 6 months (**A**), 12 months (**B**), 3 years (**C**), 5 years (**D**), 10 years (**E**), and 19 years (**F**). (Photos shown with permission of the patient and parents).

Kabuki syndrome (KS) (Niikawa–Kuroki syndrome) is a rare, congenital disorder that was first recognized as a distinct entity by two independent groups of clinicians in 1981 [1,2]. KS has been reported across all ethnicities [3,4] at an estimated frequency of 1:32,000 to 1:86,000 [5,6]. Five main cardinal features are reported to be most prevalent: (1) characteristic facial gestalt (e.g., arched eyebrows with lateral sparsening, eversion of the lateral third of the lower eye lid, long palpebral fissures, hypoplastic columella, prominent ears, cleft lip and/or palate) (Figure 1); (2) skeletal abnormalities; (3) dermatoglyphic abnormalities; (4) mild-to-moderate intellectual disability; (5) postnatal growth deficiency [5].

We now recognize that the clinical phenotype of KS follows a highly variable and continuous spectrum that additionally includes cardiac, renal, genitourinary and/or inner ear structural defects, dental anomalies, developmental delays, hypotonia, feeding difficulties, hearing loss, as well as involvement of the immune, neurologic, ophthalmologic, gastrointestinal, and endocrine systems [4,5,7]. Aspects of the clinical phenotype in KS may be accentuated in different ethnicities, as recently reported from Italy, China and Korea [8,9,10].

Updated international consensus diagnostic criteria were recently published [8]. Historically, the diagnosis of KS depended on the recognition of the characteristic facial gestalt. These features evolve over time and may make a clinical diagnosis of KS more challenging in older individuals (Figure 1).

The identification of the molecular basis of KS [9,10] allows for targeted genetic testing of individuals who may exhibit the classic phenotype of KS and for next generation sequencing (NGS) (e.g., gene panel, exome or genome) of individuals who may demonstrate certain aspects of KS. Heterozygous pathogenic variants in *KMT2D* are associated with autosomal dominant KS type 1 (KS1, MIM #147920) whereas heterozygous or hemizygous variants in *KDM6A* are associated with X-linked dominant KS type 2 (KS2, MIM #300867).

The vast majority of individuals (>80%) with a clinical diagnosis of KS harbor pathogenic variants in the coding regions of *KMT2D*, whereas a much smaller number (6–10%) harbor pathogenic variants in the coding regions of *KDM6A* [10,11,12,13]. Pathogenic variants in deep intronic or regulatory sequences of either gene have, to our knowledge, not been reported. Additional genes have been implicated in the etiology of KS-like phenotypes including *RAP1A/RAP1B, HNRNPK* and *ZMZ1* [14,15,16]. Likely, other genes associated with a KS-like phenotype do exist.

The majority of pathogenic variants in both *KMT2D* and *KDM6A* are, de novo, either associated with loss-of-function (nonsense, frameshift, splice site) resulting in haploinsufficiency or with missense variants, which may pose a challenge for variant classification [9,10]. Mosaicism and familial variants have been reported in a few affected individuals with KS [17,18,19,20,21,22,23,24,25]. Widespread use of NGS has led to the identification of additional KMT2D-related disorders in addition to KS: Individuals with missense variants in a highly conserved region of 54 amino acids in exons 38 or 39 of *KMT2D* present with a unique clinical phenotype that includes choanal atresia, athelia, thyroid abnormalities, abnormal pubertal development, and short stature [26,27]. *KMT2D* variants may also be associated with isolated alobar holoprosencephaly [28,29]

*KMT2D* encodes a lysine-specific methyltransferase, responsible for post-translational histone 3 lysine 4 (H3K4) mono-, di- and tri-methylation, which is exclusively associated with actively transcribed genes [4,30]. *KDM6A* encodes an X-linked H3K27 demethylase that removes repressive epigenetic marks and interacts with *KMT2D* in regulating gene expression in the activating signal cointegrator-2 -containing complex (ASCOM) complex [31]. Transcriptional regulation is a highly specialized and temporally orchestrated process during early embryogenesis and development, ensuring the correct time and location of gene expression. This temporal and spatial coordination of transcription determines cell fate, cell-cycle progression, stem cell function, and ultimately normal embryogenesis. The complexity and importance of perfectly concerted transcriptional control necessitate a number of regulatory mechanisms, including chromatin and histone modifications. While histone modifications are conserved and broadly used mechanisms in transcriptional regulation, many of these enzymes act very specifically on their target substrates in mammals.

This review aims to appraise our current knowledge of Kabuki syndrome with specific attention to clinical presentation and associated genotypes to identify gaps in knowledge and unmet patient needs.

## 2. Materials and Methods

A literature search was conducted using EMBASE and Ovid MEDLINE databases by two independent individuals (KB and MT). Search terms and inclusion and exclusion criteria are detailed in Table 1. Search dates were from 1981 to April 2021. All search terms were combined using the operator “OR” (e.g., “Kabuki syndrome” OR “Niikawa–Kuroki syndrome”).

## 3. Results

### 3.1. Literature Search

A total of 2418 citations matching the search terms were identified (EMBASE 1270; Ovid MEDLINE 1148). After removal of duplicates and evaluation of abstracts for exclusion and inclusion criteria (Table 1), 152 citations were included in the final analysis.

### 3.2. Demographics

A total of 1369 individuals with KS were identified in the published literature. When gender was reported (n = 582), 43% were male, and 57% were female. The average age at the time of publication was 9.97 years (median 9 years, range 0–45 years). Reported ethnicity (n = 475) was as follows: Scandinavian (18%), US-American (14%), British (14%), Italian (10%), Chinese (8%), Brazilian (5%), Korean (4%), Australian (4%), Turkish (4%), French (3%), German (3%), Japanese (3%), Canadian (2%), Czech (2%), Thai (2%), Welch (1%). Case reports from Taiwan, Spain, Ireland, Palestine, Iran, Morocco, Egypt, Columbia, and Ecuador were also identified.

### 3.3. Genotype

A total of 1174 individuals with pathogenic or likely pathogenic variants in *KMT2D* were identified. The remaining 195 individuals carried a clinical diagnosis of KS without molecular confirmation. The majority of *KMT2D* variants were truncating (nonsense: n = 241 or frameshift: n = 137), followed by missense (n = 163), small deletions (n = 95), and splice site variants (n = 66). When recorded (n = 718), the relative frequency of variants correlated with the size of the exon and was the highest for exons 39 (20% of variants), 48 (15%), 31 (11%), 34 (7%), 11 (5%), and 10 (5%) of *KMT2D*. Five mosaic *KMT2D* cases were identified with reported levels of mosaicism of 68%, 40%, 32% and 37% in blood [17,24,25]. One study did not specify the level of mosacism [17].

A total of 89 individuals with pathogenic or likely pathogenic variants in *KDM6A* were identified. When described (n = 56), the majority of *KDM6A* variants were truncating (nonsense: n = 15, or frameshift: n = 10), followed by splice site (n = 11), missense (n = 10), large deletions (n = 3), small insertions (n = 3), indel (n = 2), and small deletions (n = 2). In a recent study which evaluated 61 pathogenic *KDM6A* variants in patients with KS type 2, truncating variants were distributed across the entire gene whereas missense variants were primarily clustered in the TRP2, TRP3, TRP 7, and Jmj-C domains [23]. One mosaic *KDM6A* case was identified; the level of mosaicism was not reported [18]

In total, 19 cases of inherited pathogenic variants from affected parents were identified including 14 inherited *KDM6A* variants (13 maternally inherited, 1 paternally inherited) and 5 inherited *KMT2D* variants (5 maternally inherited) [19,20,21,22,23,25,32]. Additional clinically diagnosed familial cases were reported [33,34,35,36]. A sex-specific pattern was observed across inherited cases of KS2, with affected mothers and daughters exhibiting a milder phenotype and affected sons displaying a more severe or “classic” phenotype. In mothers, typical facial features were less apparent and intellectual disability was generally mild or absent [19,20,21]. In the paternally inherited case of KS2, the father and daughter were similarly affected [37]. Prenatal and antenatal history is sparse for most of these cases. A complex congenital heart defect was identified antenatally in one maternally inherited case of KS2 [21]. Additionally, one family had co-inheritance of generalized epilepsy with febrile seizures plus (GEFS+) and KS2 [22]. In familial KS1, there is marked clinical heterogeneity within families without obvious sex-specific differences.

### 3.4. Facial Gestalt

The cardinal facial features associated with KS include long palpebral fissures; eversion of the lateral third of the lower eyelid; arched and broad eyebrows with a sparse lateral third; a broad/depressed nasal tip with short columella; and large, prominent, or cupped ears [11]. Dysmorphic facial features are most apparent in early childhood (between 3 and 12 years) [8], and to less extent in neonates, infants, and adults [13,38] (Figure 1). Though facial dysmorphism is considered a cardinal feature of KS, patients with KS2 may be less likely than patients with KS1 to have typical facial features [23].

### 3.5. Congenital Heart Defects

The reported frequency of congenital heart defects in KS varies, ranging between 28 and 80%; most are diagnosed prenatally or at an early age [7,39] and primarily involve the left ventricular outflow tract, including the atrial and ventricular septa [7,24]. In descending order of prevalence, ventricular septal defects, coarctation of the aorta, atrial septal defects, bicuspid aortic valve, patent ductus arteriosus, and hypoplastic left heart syndrome were most commonly reported [7,10,40,41,42,43]. The prevalence of cardiovascular anomalies may be higher in KS2 compared with KS1 [23].

### 3.6. Immunologic Profile

Immune deficiency and autoimmune disorders manifest in 60–73% of KS patients. Hypogammaglobulinemia occurs in up to 58% and IgA deficiency in up to 80% [44,45,46,47,48,49,50]. Like common variable immunodeficiency (CVID), immunoglobulin levels may be normal in childhood but later become abnormal [51], and autoimmune manifestations may increase with age [52]. In a large KS registry, the prevalence of infection, hypogammaglobulinemia, immune thrombocytopenia, autoimmune hemolytic anemia, thyroiditis, and vitiligo were similar between children (<9 years), teens (9–18 years), and adults (>18 years); however, the prevalence of autoimmune disease was statistically significantly higher in adults compared to children and teens [52]. Chronic otitis media, a sequela of childhood immunodeficiency, is common in children with KS, occurring in up to 40% of individuals. In rare cases, severe otitis media can result in deafness [53]

Autoimmune disorders typically manifest in late childhood between 4 and 13 years [54]. The most frequent autoimmune manifestations include immune thrombocytopenic purpura (with or without concurrent hemolytic anemia or autoimmune neutropenia) [54,55], vitiligo [56], autoimmune thyroiditis [57], type 1 diabetes [6,58,59,60], and type 3 membranous glomerulonephritis [61]. Interestingly, selective IgA deficiency (common in KS), may be causally related to the development of type I diabetes [59,62,63]. Inflammatory bowel disease has also been reported [64].

Rare cases of premature death have been reported in severely immunologically impaired patients [46,51,52,58,65]. In one study, three of the five deaths were secondary to immunopathological complications including acute bronchitis (n = 2) and acute hemolysis secondary to chronic Evans syndrome (n = 1) at <1, 2, and 24 years of age, respectively [52].

### 3.7. Brain and Neurologic Manifestations

Congenital central nervous system malformations and functional neurological abnormalities are common. Muscular hypotonia is highly prevalent (51–98%) and may lead to long-term sequelae including developmental delays and oromotor dysfunction [66]. Emerging evidence suggests that hypotonia may be due to a primary defect in skeletal muscle [67]. Dysarthria is not uncommon (10–26%) and may be underestimated [68]. Vestibular dysfunction also contributes to gross motor delay [69]. The estimated prevalence of epilepsy is 17%, with the age of onset ranging from infancy to adolescence [70,71,72]. Partial seizures involving the frontal and temporal regions manifesting with focal motor deficits are well-described [71,73,74,75,76,77]. No relationship between epileptogenic zone and polymicrogyria has been established [74,75,78].

Structural central nervous system anomalies may have an ethnic association; most cases of cortical dysplasia were reported in Caucasian individuals, although reporting biases cannot be excluded [78]. The reported spectrum of congenital brain abnormalities continues to expand. Recently, a case of lobar holoprosencephaly was described [79].

### 3.8. Cancer

The incidence of cancer and whether individuals with KS have an increased predisposition for malignancy is unknown. Somatic mutations in the histone methyltransferase *KMT2D* are frequently implicated in tumorigenesis and depending on the biologic context, this methyltransferase may exert either tumor suppressive or promoting functions [80,81]. It has been proposed that patients with germline variants in *KMT2D* may be at an increased risk for developing cancer due to somatic second hit mutations [81]. Despite this, less than twenty patients with Kabuki syndrome and malignancies, mostly affecting soft tissues or the hematological system, have been reported. Solid and soft tissue neoplasms reported in KS and age at diagnosis include: Wilms tumor, 3 years [82]; neuroblastoma, 6 months [83]; low-grade fibromyxoid sarcoma, 11 years [84]; synovial sarcoma, 16 years [85]; aggressive desmoid fibromatosis, 10 years [81]; hepatoblastoma, 6 years [83]; hepatocellular carcinoma, 15 years [86]; giant cell fibroblastoma, 12 years [87]; spinal ependymoma, 23 years [88]. Hematologic malignancies and age at diagnosis include Hodgkin lymphoma, 34 years [89]; acute lymphocytic leukemia, 1 year [90]; Burkitt lymphoma, 3 and 5 years, respectively [91,92]. Notably, Epstein-Barr virus (EBV)-positive Burkitt’s lymphoma is more commonly seen in immunocompromised patients, perhaps explaining its relationship with KS [93,94]. Pilomatrixomas, a benign tumor of the hair cell matrix, are also infrequently reported [95,96].

### 3.9. Endocrinopathies

Endocrine dysfunction is common in KS. Postnatal growth restriction occurs in 35–85% of individuals and is far more common than growth hormone deficiency [5,97,98,99]. Recent attention has focused on persistent hyperinsulinemic hypoglycemia, which is likely underdiagnosed or underreported in KS [18,100]. If untreated, hyperinsulinemic hypoglycemia can lead to developmental delay and permanent neurologic damage [18]. Persistent hypoglycemia in infancy is attributed to pituitary hormone deficiency, growth hormone deficiency, adrenal insufficiency, and, more notably, dysregulated insulin secretion by the pancreatic β-cells. Persistent hypoglycemia may be more common in KS2, suggesting a possible genotype–phenotype correlation [12]. Supporting this, inhibition of *KDM6A* increased the release of insulin from pancreatic islet cells in murine models [101].

Two cases of isolated central diabetes insipidus (DI) are described in the literature [102,103]. Although congenital brain malformations can cause central DI, most cases are idiopathic [103]. Of these case reports, one patient had an abnormal pituitary gland and stalk on MRI.

In KS, the risk for diabetes mellitus type II may be as high as 20% in early adulthood [104]. In such cases, type II diabetes is usually comorbid with obesity.

### 3.10. Genitourinary Anomalies

In total, 30–40% of individuals with KS have a genitourinary anomaly [66]. Both renal and urogenital anomalies are common; the most common renal malformations are horseshoe kidneys and renal hypoplasia [105]. In males, cryptorchidism and hypospadias are the most prevalent urogenital anomalies [13,106,107]. Rare cases of other anomalies including ureteric and renal duplication, ectopic kidneys, renal agenesis, hypoplastic labia are reported [40,87,108,109]. Renal dysfunction and failure are rare, occurring in a few isolated cases [97,106,110,111,112]. Most cases of severe renal insufficiency are secondary to congenital renal dysplasia. Renal anomalies can increase susceptibility to urinary tract infections and, less frequently, renal calculi. Interestingly, patients with KS2 may have a higher prevalence of genitourinary anomalies than those with KS1 overall but a lower prevalence of kidney and renal tract anomalies [23].

### 3.11. Ophthalmologic Abnormalities

Ocular abnormalities occur in 38–72% of KS patients [113,114]. In addition to cardinal features (e.g., long palpebral fissures, lower eyelid eversion, and epicanthus), ptosis, epiblepharon, and centurion eyelid syndrome with consecutive epiphora are observed in 10–20% of individuals [115]. Refractive errors, notably astigmatism, may occur in up to 90% of patients [115]. Microphthalmia, anophthalmia, and coloboma (MAC) spectrum is the third most common manifestation in about 3.2% of cases [88]. In a large cohort of patients with KS2, nystagmus was reported in 11% of patients [23].

### 3.12. Gastrointestinal Involvement

Feeding difficulty and reflux are highly prevalent in KS [40], but congenital gastrointestinal anomalies are rare, with an incidence of about 5% [3]. The prevalence of liver disease ranges from 2–21%.

### 3.13. Reproductive Health

There is a paucity of information regarding fertility and reproductive health pertaining to KS. Familial transmission of KS both to and from affected males and females has been reported [19,20,21,22,37]. Endocrine dysregulation can interfere with fertility, but the extent and implication of this are not adequately discussed in the existing literature [116]. Both patients and clinicians would benefit from further research in this area.

### 3.14. Prenatal and Perinatal History

There is a paucity of data concerning prenatal and perinatal complications of KS. One study reported abnormal second and third trimester ultrasounds and quad screens in 69% and 44% of cases, respectively [117]. Polyhydramnios is reported in 25–41% of pregnancies [117,118,119] compared to 0.5–2% in the general population [120,121]. It is plausible that craniofacial anomalies or functional impairment in swallowing, both well-described features of KS, contribute to this finding [117].

### 3.15. Growth and Feeding

Postnatal growth restriction (60–83%) and short stature (31–81%) [5,97] are common in KS. Birth parameters are typically normal, but failure to thrive and growth restriction become apparent in infancy, likely secondary to feeding problems and hypotonia, resulting in poorly coordinated sucking and swallowing [40,97]. Severe feeding difficulty in infancy necessitates nasogastric tube or gastrostomy tube placement in 65–74% of infants [40,113]. Premature birth, growth hormone deficiency, delayed bone age, aspiration pneumonia, congenital heart defects, and palatal anomalies [122] also contribute to delayed or abnormal growth but do not entirely explain the growth restriction [105]. In KS2, sex-specific differences in growth parameters have been described; in one study, males had shorter birth lengths and significantly shorter stature at last examination compared to their female counterparts [23]. There is limited research evaluating the impact of recombinant human growth hormone (rh-GH) therapy in children with KS. Several small studies have shown promising results, demonstrating statistically significant increases in linear growth following at least one year of rh-GH therapy, without negatively impacting cardiovascular health [123,124,125]. Without growth hormone therapy, adult height may fall anywhere from −5.57 SD and −1.08 SD below the mean for healthy controls, with females more severely affected than males [126].

The natural history of KS is poorly characterized. In one Japanese study, childhood height ranged from −2.1 to −5 SD below the mean for healthy controls; 27% of children had height within the normal range; none were greater than +0.5 SD above the mean [5]. In another study, the average birth length was −0.14 SD and −0.11 SD below the mean for males and females, respectively. Growth restriction became more apparent by one year of age and was maintained into adulthood [122]. KS1-specific growth charts were recently developed to establish normative growth parameters [127].

Interestingly, up to 50% of individuals with KS will become overweight or obese in late childhood or adolescence [6,23,40,97,127]. In one study, BMI was in the overweight to obese range in 57% of patients over 5 years old [6]; 75% of these children failed to thrive in infancy. Unsurprisingly, obesity in adolescence is associated with significant comorbidities and can exacerbate pre-existing medical issues, such as patellar dislocation [128], which is more common in older children with KS, particularly females with hypermobile knee joints. Patients with KS may also have an inherent predisposition for developing hypertension, even in the absence of other metabolic risk factors, due to premature atherosclerosis [129] and growth hormone deficiency, which is associated with endothelial dysfunction, increased intimal thickness, and reduced aortic elasticity [7].

### 3.16. Language and Development

Developmental milestones are often delayed in children with KS. Cognitive impairment, hypotonia, skeletal anomalies, congenital heart defects, hearing loss, and gastrointestinal anomalies can cause delays in speech and language development, feeding and swallowing, walking, sitting upright, and toilet training [25,119,130,131,132,133]. Conductive and sensorineural hearing loss, in particular, are associated with poor verbal outcomes [119].

In one study, the average age of acquisition of developmental milestones was as follows [119]: sitting unassisted, 11 months; walking without assistance, 20 months; single word utterance, 21 months. Language development was uniformly delayed; only one patient acquired their first word at a developmentally appropriate time.

Language acquisition varies in KS [134,135]. Although most children will eventually speak in complete sentences, isolated case reports describe children without a single word or simple sentence development until upwards of 10 years [133,136]. Patients may be nonverbal at the extreme end of the spectrum [134,137]. Motor and language development do not always correlate; some individuals may have significant motor impairment with relatively strong language skills and vice versa.

No clear pattern of psychomotor development has emerged, and data are limited to sporadic case reports [1,2,36,134,138,139]; however, some genotype–phenotype hypotheses have emerged. Haploinsufficiency of *KDM6A* is similarly associated with more severe psychomotor developmental delays [134]. In KS2, males may exhibit more severe neurodevelopmental issues, whereas females with KS2 display a more variable neurodevelopmental profile, possibly due to differences in X-chromosome inactivation [23]. Patients with protein-truncating mutations in *KDM6A* versus protein-altering variants may also display more severe intellectual disability [23].

### 3.17. Cognitive Profile

Intellectual disability (ID) is one of the cardinal features of KS, presenting in up to 90% of cases [98,99]. ID is generally designated with intelligence quotient (IQ) scores of less than 70, which falls into the “very low range of functioning” (bottom 2%) of age-matched individuals [140]. In KS, intellectual disability is typically mild to moderate; average IQ is in the upper-50s or low-to-mid 60s [25,141,142] with a range of 25–109 [17]. Up to 92% of individuals with KS will have an IQ of 80 or less [143]. IQ scores in the low 40s represent the lower bound of performance [17,57,141,144,145].

Severe ID is rare and only described in a few cases [12,17,76,146]. Individuals with KS typically possess relative strengths in verbal reasoning and working memory with excellent long-term memory of faces, music, lyrics, events, and dates [6]. Age-appropriate planning, cognitive flexibility, and social cognition are also observed [147]. Conversely, relative deficiencies in visual-spatial skills, processing speed, and nonverbal reasoning are described [17,25,130,147]. Further insight into difficult cognitive tasks reported in KS by parents include reading a map, following directions to a location, and math problems [148].

### 3.18. Communication, Speech, and Language

A variable pattern of oromotor, speech, and language deficits reflects neurologic, orofacial, structural, hearing, and cognitive impairment.

Receptive and expressive language delays present across multiple language sub-domains (e.g., semantics, syntax, morphology, and pragmatics) [68,119]; however, a deficit in one language domain does not predict disordered development in another [149]. Articulation and resonance can also be affected [68,150]; speech is often described as “thick, slurred, and indistinct” [133] and resonance as “hyper-nasal” [68,151], likely secondary to poor oromotor coordination and hypotonia [149,150]. Dysarthria, characterized by imprecise consonants, harsh vocal quality, hypernasality, and slowed rate of speech, is common. Children with otherwise normal cognition and appropriate expressive/receptive vocabulary may use phonological processes to simplify words or demonstrate poor morphosyntactic (e.g., ‘word endings’ such as -ing, plural -s) abilities [132].

Some studies report improvements in speech, language, and oromotor function with age; however, others conclude the opposite, noting worsening language impairment over time [74,152]. Regardless, most individuals with KS will have communication deficits persisting into early childhood or adolescence [68].

### 3.19. Behavioral Phenotype

Autistic-like behavior, hyperactivity, inattention, impulsivity, self-mutilation, sleep disturbances, multiple phobias, emotional dysregulation, impairment of adaptive skills, difficulty with communication and peer interactions, poor eye contact, and anxiety disorders are among the diverse behavioral phenotypes associated with KS [6,10,11,12,56,58,138,153,154,155]. Though children may exhibit autistic-like behaviors, diagnostic criteria for autism are met in a minority of cases [11,138].

Severe behavioral phenotypes (e.g., aggression, oppositional behavior) are rare, occurring only in patients with severe cognitive deficits [130]. Children with KS generally behave as expected for their chronological age and demonstrate age-appropriate maladaptive behavior [130].

More consistently, children with KS are socially outgoing, talkative, and affectionate, with good socialization skills and affable nature [6,130]. A small survey of adolescents with KS reported a range of emotional impacts that included anxiety, sadness, frustration, and feeling different from other people [89].

## 4. Conclusions

A broad and continuous spectrum of clinical phenotypes is associated with KS, with notable variability between affected individuals. Advancements in genetic sequencing improve our understanding of this disease and elucidate the role of *KMT2D* and *KDM6A* in transcriptional regulation, growth, and development. Current literature lacks consistent clinical characterization of KS, posing challenges in drawing meaningful genotype–phenotype correlations. Limited long-term follow-up similarly restricts our understanding of the natural history of the disease. Unfortunately, clinical management is fragmented and remains largely symptom oriented.

This review holistically examines KS and details the natural history as described in current literature to guide the surveillance and expectant management of KS (Table 2, Table 3 and Table 4). Clinicians, caregivers, and affected individuals would benefit from an improved understanding of the disease burden and impact on quality of life, the adult phenotype, and the development of standard-of-care guidelines.

## Figures and Tables

**Table 1:** Search terms, inclusion, and exclusion criteria.

Search Terms	Inclusion Criteria	Exclusion Criteria
▪ Kabuki syndrome ▪ Niikawa–Kuroki syndrome ▪ Kabuki make-up syndrome ▪ MLL2 ▪ KMT2D ▪ KDM6A	▪ English language (or available translation) ▪ Human Studies ▪ Full text available ▪ Clinical and/or genetic information about KS	▪ Duplicate study ▪ Limited relevance ▪ Primary topic unrelated to KS

KS = Kabuki syndrome.

**Table 2:** Recommended initial evaluation by organ system.

System ^1^	Evaluation	Comment
**Growth**	Measurement of height, weight, and head circumference	While infants may have FTT, adolescents and adults may have obesity. KS-specific growth charts available
**Ophthalmologic**	Ophthalmology evaluation	For assessment of strabismus, refractive error, ptosis, and corneal abnormalities
**Hearing**	Baseline audiology evaluation	To assess for conductive and/or sensorineural hearing loss
**Mouth**	Directed evaluation of the palate for palatal anomalies	Consider referral to a craniofacial specialist if palatal anomalies are suspected
	Consider dental evaluation for those over 3 years of age	
**Cardiac**	Echocardiogram with visualization of the aortic arch	To assess for congenital heart defects, including coarctation of the aorta
	Consider EKG	If arrhythmia is suspected
**Respiratory**	Consider chest radiographs to assess for diaphragmatic eventuation	In those with respiratory issues, chronic cough, or recurrent pneumonia
**Gastrointestinal/Feeding**	Asses nutritional status, feeding, GERD	Consider assessment by a feeding team and/or a VFSS for those with suspected dysphagia
**Genitourinary**	Baseline renal ultrasound	To evaluate for renal anomalies and hydronephrosis
	Physical examination for hypospadias and/or cryptorchidism in males	
**Musculoskeletal**	Consider radiographs of the spine in those with scoliosis	To assess for vertebral anomalies
**Endocrinologic**	Assessment for hyperinsulinism	In neonates and infants with persistent hypoglycemia
	Assessment for hypothyroidism and growth hormone deficiency	In those with an abnormal growth velocity
**Immunologic**	T cell count, T cell subsets, and serum immunoglobulin levels at the time of diagnosis or at age one year (whichever comes later)	Referral to immunology if immunological studies are abnormal or there is a history of recurrent infections
**Neurologic**	EEG	In those with suspected seizures
	Brain MRI	To evaluate for a structural brain malformation in those with seizures
		To evaluate for Chiari I malformation in those with suggestive symptoms
**Psychiatric/behavioral**	Neuropsychiatric evaluation	To include screening for the presence of behavioral problems, including sleep disturbances, ADHD, anxiety, and/or traits suggestive of ASD for individuals age >12 months
**Miscellaneous/Other**	Developmental assessment	To include motor, speech/language evaluation, general cognitive, and vocational skills
	Consultation with a clinical geneticist and/or genetic counselor	Medical home, care coordination, molecular diagnosis and counseling

^1^ Adapted with permission from *GeneReviews*^®^; Adam MP, Hudgins L, Hannibal M. Kabuki Syndrome. 1 September 2011 [Updated 15 July 2021]. In: Adam MP, Ardinger HH, Pagon RA et al., editors. *GeneReviews*^®^ [Internet]. Seattle (WA): University of Washington, Seattle; 1993–2022. Available from: https://www.ncbi.nlm.nih.gov/books/NBK62111/ (accessed on 29 April 2022). KS = Kabuki syndrome; FTT = failure to thrive; EKG = electrocardiogram; GERD = gastroesophageal reflux disease; VFSS = videofluoroscopic swallow study; EEG = electroencephalogram; MRI = magnetic resonance imaging; ADHD = attention-deficit/hyperactivity disorder; ASD = autism spectrum disorder.

**Table 3:** Health surveillance by organ system.

System ^1^	Evaluation	Frequency
**Growth**	Measurement of at least height and weight	At each appointment
**Ophthalmologic**	Ophthalmology or optometry to assess vision	At least annually
**Hearing**	Hearing assessment	At least annually
**Musculoskeletal**	Clinical evaluation for scoliosis	At each appointment until skeletal maturity
**Endocrinologic**	Thyroid function tests	Every 2–3 years
**Immunologic**	Assessment of complete blood count, immunoglobulin levels, flow cytometry?	Every 2–3 years
**Miscellaneous/Other**	Monitor developmental progress and educational needs	At each visit during childhood and adolescence

^1^ Adapted with permission from *GeneReviews*^®^; Adam MP, Hudgins L, Hannibal M. Kabuki Syndrome. 1 September 2011 [Updated 15 July 2021]. In: Adam MP, Ardinger HH, Pagon RA et al., editors. *GeneReviews*^®^ [Internet]. Seattle (WA): University of Washington, Seattle; 1993–2022. Available from: https://www.ncbi.nlm.nih.gov/books/NBK62111/ (accessed on 29 April 2022).

**Table 4:** Treatment of manifestations by indication.

Manifestation ^1^	Treatment	Considerations/Other
**Strabismus, refractive error, ptosis, lagophthalmos**	Standard treatment per Ophthalmology	
**Hearing loss**	Consideration of pressure equalizing tubes in those with conductive hearing loss	Referral to an ENT specialist and audiologist
	Hearing aids may be considered for those with sensorineural hearing loss	
**Cleft lip and/or palate**	Standard treatment	Management through a specialized Craniofacial clinic is ideal
		The palate may be shorter, which can lead to velopharyngeal insufficiency after typical cleft repair
**Dental anomalies**	Orthodontic referral if hypodontia or significant malocclusion are noted	
**Congenital heart defects and/or arrhythmia**	Standard treatment per Cardiology	It is unclear whether there is an increased risk for aortic aneurysm; however, if catheterization or angioplasty is being considered, a potential increased risk of aortic aneurysm should be communicated to the treating team
**Feeding difficulties/GERD**	Standard treatment, which may include thickening feeds and appropriate positioning after meals in infants and toddlers	Pharmacologic treatment for GERD may be considered
	Consideration of gastrostomy tube	In those with severe feeding difficulties and/or poorly coordinated suck and swallow
**Chronic diarrhea**	Referral to a Gastroenterology specialist	Consider evaluation for malabsorption and/or celiac disease
**Hypospadias/cryptorchidism**	Standard treatment per Urology	
**Hyperinsulinism and hypothyroidism**	Standard treatment per Endocrinology	
**Short stature**	Consideration of growth hormone therapy	
**Recurrent infections**	Intravenous immunoglobulin (IVIG) therapy may be considered in those this documented immunoglobulin deficiency	Referral to Immunology
**Seizure disorder**	Standard antiepileptic treatment per Neurology	
**Premature thelarche**	No treatment is warranted if there are no other signs of premature puberty	
**Need for anesthesia**	Care in positioning during intubation due to joint laxity, which can affect the cervical spine	Education regarding potential structural airway anomalies that could make intubation difficult

^1^ Adapted with permission from *GeneReviews*^®^; Adam MP, Hudgins L, Hannibal M. Kabuki Syndrome. 1 September 2011 [Updated 15 July 2021]. In: Adam MP, Ardinger HH, Pagon RA et al., editors. *GeneReviews*^®^ [Internet]. Seattle (WA): University of Washington, Seattle; 1993–2022. Available from: https://www.ncbi.nlm.nih.gov/books/NBK62111/ (accessed on 1 September 2022).

## Data Availability

Not applicable.
